# Safe Laparoscopic Cholecystectomy in Mirror-Image Anatomy: Acute Biliary Pancreatitis and Acute Calculous Cholecystitis in Situs Inversus Totalis

**DOI:** 10.7759/cureus.112279

**Published:** 2026-07-08

**Authors:** Brayan A Flores-Zepeda, Vicente Nieto-López, Jorge O Beltrán-Delgado, José E Soto-Valle, Ricardo A Valdez-Valdez

**Affiliations:** 1 General Surgery, Center for Research and Teaching in Health Sciences, Autonomous University of Sinaloa, Culiacan Civil Hospital, Sinaloa, MEX

**Keywords:** acute biliary pancreatitis, calculous cholecystitis, critical view of safety, laparoscopic cholecystectomy, mirror-image anatomy, situs inversus totalis

## Abstract

Situs inversus totalis (SIT) is a rare congenital laterality anomaly characterized by the complete mirror-image transposition of the thoracic and abdominal viscera. Although it is usually asymptomatic, the occurrence of acute biliary disease poses significant diagnostic and surgical challenges because the atypical location of pain and reversed anatomy may complicate clinical assessment, interpretation of imaging studies, and laparoscopic planning. We present the case of a patient with acute biliary pancreatitis associated with acute calculous cholecystitis in the setting of SIT, whose management required individualized diagnostic evaluation and surgical planning tailored to the mirror-image anatomy. A 20-year-old woman with a known diagnosis of SIT presented with a three-day history of severe epigastric pain radiating to the left upper quadrant and back, accompanied by nausea and vomiting. Laboratory evaluation demonstrated markedly elevated pancreatic enzyme levels, mild hyperbilirubinemia, and elevated liver transaminases. Abdominal ultrasonography revealed cholelithiasis and findings suggestive of SIT. Contrast-enhanced computed tomography confirmed dextrocardia, complete visceral transposition, acute pancreatitis with peripancreatic inflammatory changes, and imaging findings consistent with acute calculous cholecystitis without biliary ductal dilatation. Based on the Revised Atlanta Classification, the patient was diagnosed with mild acute biliary pancreatitis. Following clinical stabilization, laparoscopic cholecystectomy was successfully performed using a mirror-image trocar configuration adapted from the conventional technique. The postoperative course was uneventful, and histopathological examination confirmed chronic cholecystitis associated with cholelithiasis. SIT is not a contraindication to laparoscopic cholecystectomy in patients with acute biliary disease; however, it requires early recognition of the mirror-image anatomy, accurate interpretation of imaging findings, and individualized surgical planning. In this setting, mirror-image trocar placement and strict adherence to the critical view of safety are essential to minimize the risk of bile duct injury. This case demonstrates that, with meticulous preoperative anatomical assessment and an individualized surgical strategy, laparoscopic management can be performed safely and effectively.

## Introduction

Situs inversus is a rare congenital laterality anomaly that may present as situs inversus totalis (SIT), characterized by complete transposition of the thoracic and abdominal viscera, or as situs inversus partialis, in which only selected thoracic or abdominal organs exhibit reversed laterality. In SIT, the heart is typically located in the right hemithorax, whereas the liver and gallbladder are positioned in the left upper quadrant, and the spleen and stomach are predominantly located on the right. Its estimated incidence ranges from 1 in 5,000 to 1 in 20,000 live births, and most affected individuals remain asymptomatic until the development of an associated congenital disorder or acquired abdominal disease [1].

Although SIT is not a disease itself, it poses significant diagnostic and surgical challenges when acute abdominal conditions occur. Biliary disease may present with epigastric or left upper quadrant pain, potentially leading to delayed diagnosis or misinterpretation of the underlying pathology. In this setting, imaging studies play a pivotal role in establishing the diagnosis, while fluorescence cholangiography has emerged as a valuable adjunct for real-time identification of the biliary anatomy during laparoscopic cholecystectomy in patients with complex anatomical variations [2].

Since the first report of laparoscopic cholecystectomy in a patient with SIT by Campos et al. in 1991, numerous case reports have demonstrated that the procedure is both feasible and safe when meticulous preoperative planning is undertaken and appropriate technical modifications are implemented [3].

The coexistence of acute biliary pancreatitis and acute cholecystitis further increases the diagnostic and therapeutic complexity of these patients. The diagnosis of acute pancreatitis is established using clinical, biochemical, and imaging criteria, whereas the Tokyo Guidelines 2018 recommend early laparoscopic cholecystectomy as the treatment of choice for patients with acute cholecystitis who are suitable surgical candidates [4]. Disease severity is classified according to the Revised Atlanta Classification [5]. The mirror-image anatomy requires modifications in operating room setup, trocar placement, and dissection strategy to maintain the principles of safe laparoscopic cholecystectomy. Several technical adaptations have been associated with favorable outcomes, although the procedure remains technically demanding, particularly in the presence of acute inflammation [6-17].

We present the case of a patient with SIT who developed acute biliary pancreatitis secondary to acute calculous cholecystitis and was successfully treated with laparoscopic cholecystectomy, highlighting the technical adaptations required to achieve a safe laparoscopic approach in this rare anatomical variant.

## Case presentation

A 20-year-old woman with a known history of SIT and no significant past medical history presented to the emergency department with a three-day history of abdominal pain. The pain was initially localized to the left upper quadrant and was unresponsive to butylscopolamine, lysine clonixinate, and ketorolac. Her symptoms were associated with nausea and multiple episodes of non-bilious, food-containing emesis, without fever.

On admission, the patient was hemodynamically stable. Physical examination revealed a mildly distended abdomen with tenderness over the left upper quadrant, a positive left-sided Murphy's sign, and localized peritoneal irritation. During evaluation by the General Surgery team, the pain was characterized as severe epigastric pain radiating to the left upper quadrant and back.

Laboratory investigations demonstrated marked elevation of pancreatic enzyme levels, mild abnormalities in liver function tests, preserved renal function, and no significant leukocytosis (Table 1).

**Table 1:** Initial laboratory findings at admission. ALT: alanine transaminase; AST: aspartate aminotransferase Created by the authors. Reference ranges correspond to the values used by the treating institution's clinical laboratory.

Parameter	Patient value	Reference range
Hemoglobin	13.4 g/dL	12-16 g/dL
Leukocytes	8.95 x 10³/µL	4-10 x 10³/µL
Neutrophils	82%	40-75%
Lipase	16,515 U/L	13-60 U/L
Amylase	1,527 U/L	28-100 U/L
AST	117 U/L	<35 U/L
ALT	212 U/L	<35 U/L
Total bilirubin	1.7 mg/dL	0.2-1.2 mg/dL
Creatinine	0.6 mg/dL	0.7-1.3 mg/dL
Glucose	68 mg/dL	70-100 mg/dL
Urea	24 mg/dL	15-45 mg/dL

**Figure 1:**
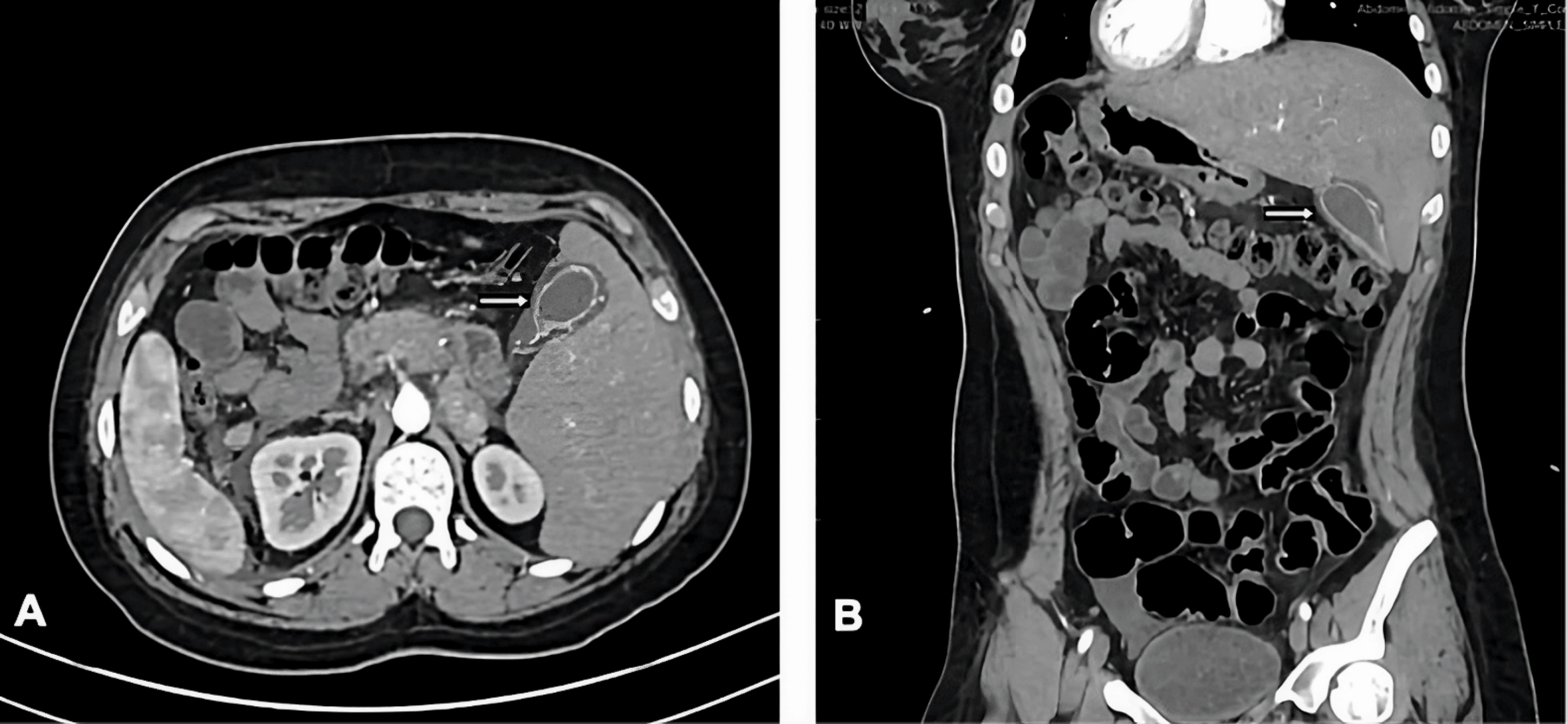
Contrast-enhanced computed tomography confirming situs inversus totalis. Contrast-enhanced computed tomography confirming situs inversus totalis. (A) Axial image demonstrating complete mirror-image transposition of the abdominal viscera. (B) Coronal reconstruction showing dextrocardia and a left-sided liver and gallbladder with inflammatory changes consistent with acute calculous cholecystitis (white arrows).

On hospital Day 1, abdominal ultrasonography demonstrated findings consistent with SIT, with the gallbladder located in the left upper quadrant containing multiple gallstones without significant gallbladder wall thickening. Contrast-enhanced computed tomography performed on hospital Day 2 confirmed dextrocardia and complete mirror-image transposition of the thoracic and abdominal viscera (Figure 1). Imaging demonstrated acute interstitial pancreatitis (Balthazar grade C; modified CT severity index, 4) with peripancreatic inflammatory changes, as well as acute calculous cholecystitis without biliary duct dilatation. Based on the Revised Atlanta Classification [5], the patient was diagnosed with mild acute biliary pancreatitis.

During the first two days of hospitalization, the patient responded favorably to conservative management, remaining hemodynamically stable, tolerating oral intake, and requiring no antibiotic therapy. The patient remained clinically stable during hospitalization, with progressive improvement in laboratory parameters, consistent with mild acute biliary pancreatitis.

**Figure 2:**
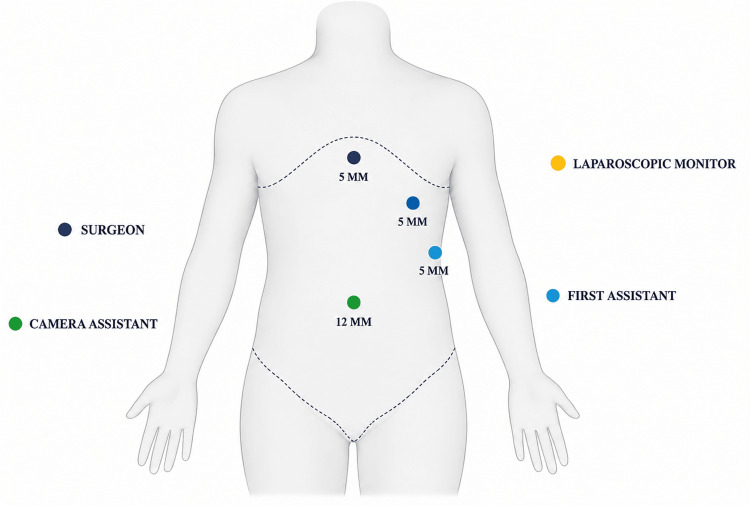
Mirror-image trocar configuration used for laparoscopic cholecystectomy in a patient with situs inversus totalis. Created by the authors using BioRender.com

**Figure 3:**
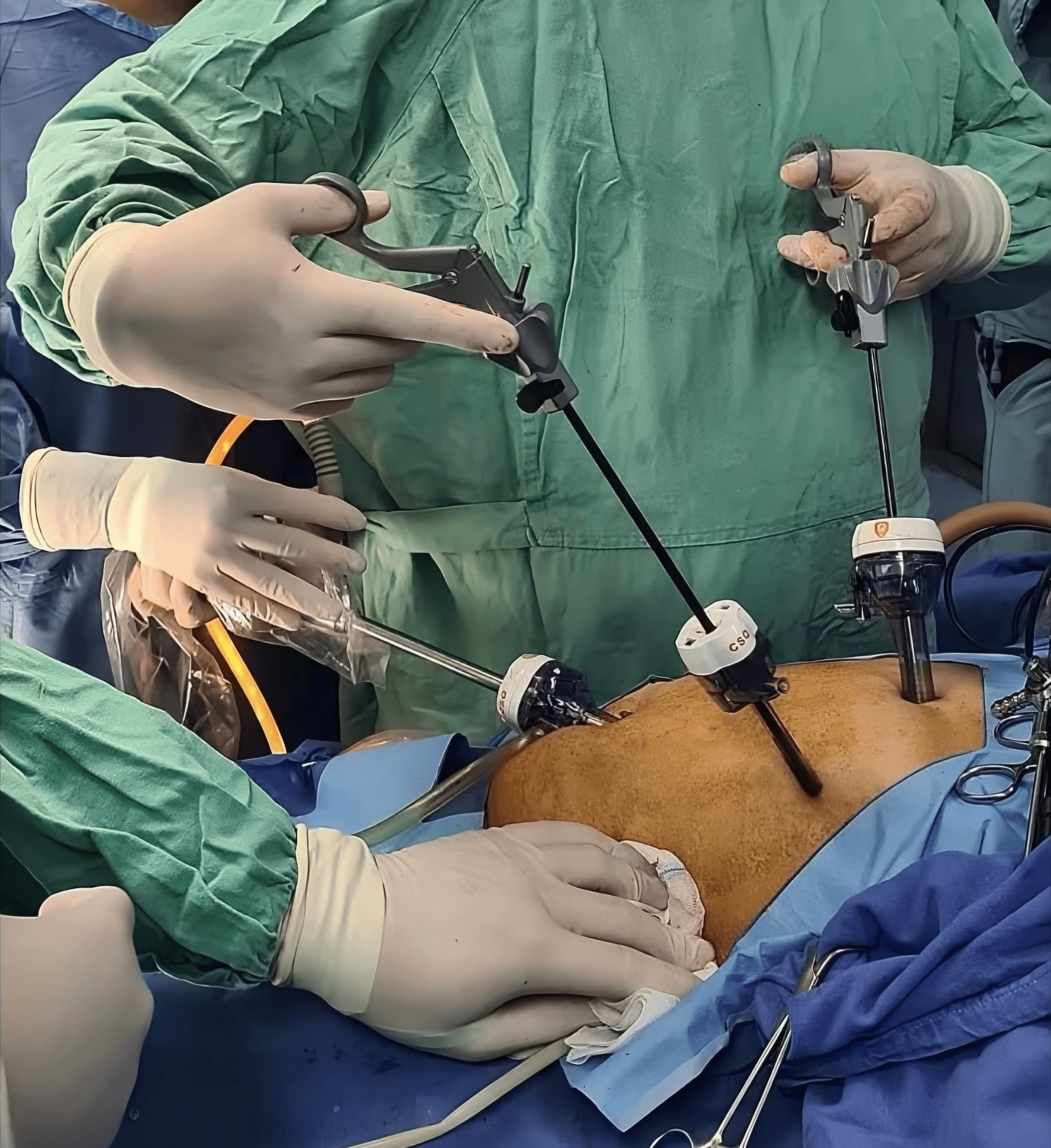
Operating room setup for laparoscopic cholecystectomy demonstrating mirror-image positioning of the surgical team and laparoscopic instruments.

Following clinical stabilization, a modified mirror-image American four-port laparoscopic cholecystectomy was performed during the index admission under general anesthesia. Pneumoperitoneum was established using the open Hasson technique through a 12-mm umbilical trocar, followed by placement of additional trocars in a mirror-image configuration adapted to the patient's reversed anatomy (Figure 2). The operating room setup was modified to accommodate the mirror-image anatomy, with the surgical team positioned accordingly (Figure 3). Intraoperative exploration confirmed SIT and a left-sided gallbladder with Parkland grade II cholecystitis and microlithiasis.

**Figure 4:**
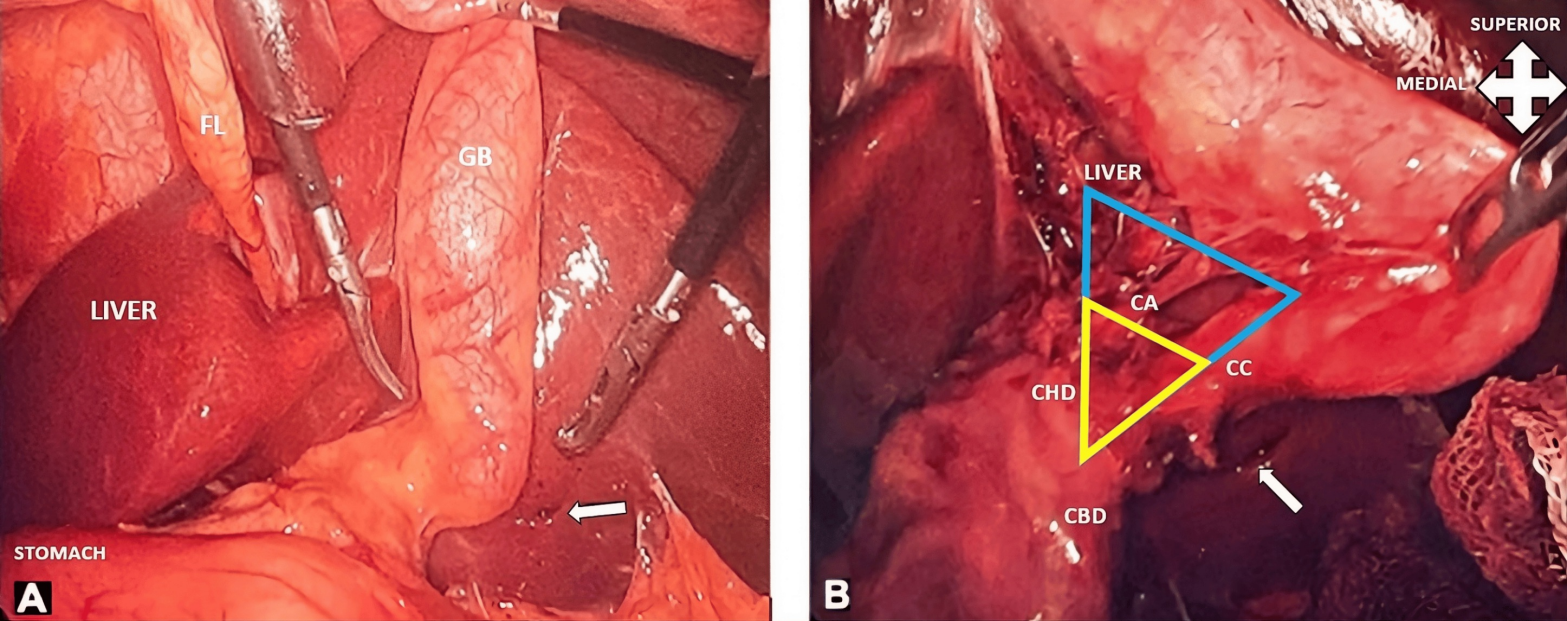
Intraoperative views during laparoscopic cholecystectomy in a patient with situs inversus totalis. (A) Initial intraoperative view prior to dissection, showing the mirror-image biliary anatomy. (B) Critical view of safety achieved before clipping and division of the cystic duct and cystic artery; the hepatocystic triangle (blue) and Calot's triangle (yellow) are outlined for anatomical reference. Arrows indicate Rouvière's sulcus in both panels. FL, falciform ligament; GB, gallbladder; CA, cystic artery; CC, cystic duct; CHD, common hepatic duct; CBD, common bile duct. Anatomical annotations added by the authors using Word (Microsoft® Corp., Redmond, WA)

Dissection was performed above Rouvière's sulcus and the proximal third of the cystic plate. Careful dissection of the hepatocystic triangle was performed (Figure 4A), followed by achievement of the critical view of safety, as described by Strasberg [16], before clipping and division of the cystic duct and cystic artery (Figure 4B). The gallbladder was then dissected from the liver bed without intraoperative complications, with an estimated blood loss of 20 mL.

The postoperative course was uneventful, and the patient was discharged on postoperative Day 1. No short- or long-term complications were observed during follow-up, and the patient remained asymptomatic, tolerated a regular diet, and demonstrated appropriate wound healing. Histopathological examination confirmed chronic cholecystitis associated with cholelithiasis.

## Discussion

SIT is a rare congenital laterality anomaly characterized by complete mirror-image transposition of the thoracic and abdominal viscera. Although SIT does not increase the incidence of cholelithiasis or acute cholecystitis, its clinical significance lies in the altered presentation of biliary disease and the diagnostic and technical challenges it poses for surgical management. Available systematic reviews consistently conclude that, with appropriate preoperative planning and technical adaptation, laparoscopic cholecystectomy can be performed safely, with outcomes comparable to those in patients with normal anatomy [6,11].

The clinical presentation represents one of the main diagnostic challenges. Whereas biliary disease typically presents with right upper quadrant pain in patients with normal anatomy, individuals with SIT often experience pain in the left upper quadrant or epigastrium because of the mirror-image arrangement of the abdominal viscera. This atypical presentation may delay diagnosis and lead clinicians to consider alternative conditions, including gastritis, peptic ulcer disease, splenic disorders, or cardiopulmonary diseases. In our patient, careful correlation of the physical examination with imaging findings enabled prompt diagnosis of acute biliary pancreatitis secondary to acute calculous cholecystitis, avoiding delays in definitive treatment [6,11].

Imaging plays a central role in confirming both the biliary pathology and the anatomical variation. Ultrasonography remains the first-line modality for detecting cholelithiasis and assessing features of acute cholecystitis, whereas contrast-enhanced computed tomography confirms visceral transposition, evaluates the severity of acute pancreatitis, and facilitates surgical planning. In the present case, both modalities were complementary and enabled accurate anatomical characterization before laparoscopic intervention [4,13].

The coexistence of acute biliary pancreatitis and SIT is uncommon and has been reported mainly in isolated case reports. Current international guidelines recommend cholecystectomy during the index admission for patients with mild acute biliary pancreatitis, as this strategy reduces recurrent biliary events without increasing morbidity or mortality. SIT does not modify this recommendation but requires meticulous preoperative assessment and a thorough understanding of the reversed anatomy. Our patient's favorable clinical course allowed definitive surgical treatment during the same hospitalization, in accordance with current evidence [13,14].

Since the first laparoscopic cholecystectomy in a patient with SIT was reported, several technical modifications have been described, including the mirror-image American technique, the French approach, modified trocar placement, the Lloyd-Davies position, and indocyanine green fluorescence cholangiography (Table 2). However, systematic reviews consistently indicate that no single configuration has demonstrated clear superiority over another. Instead, procedural success depends primarily on the surgeon's experience, ergonomic adaptation, and careful preoperative planning [6,11].

**Table 2:** Comparative table of laparoscopic techniques described for cholecystectomy in situs inversus totalis. Summary of operative positioning, trocar configuration, ergonomics, advantages, limitations, and preferred indications. ICG: indocyanine green; SILC/SILS: single-incision laparoscopic cholecystectomy; CVS: critical view of safety

Technique	Surgeon position	Port configuration	Dominant-hand ergonomics	Advantages	Limitations	Recommended use	Key references
Mirror-image American 4-port technique	Primary surgeon on patient's right; assistant on left	12-mm umbilical camera; 5-mm epigastric/subxiphoid; 5-mm left midclavicular; 5-mm left anterior axillary	Familiar to most right-handed surgeons, but reversed spatial orientation may be awkward	Most commonly reported, reproducible, good triangulation	Instrument crossing or arm torsion may occur	Standard option for uncomplicated cases	[1,6,7,11]
Modified mirror-image American technique (lowered left midclavicular port)	Primary surgeon on patient's right	Same 4-port layout, with the left midclavicular working port placed ~5 cm lower for ergonomics	Improves comfort for right-handed dissection in mirror anatomy	Reduces arm twisting; preserves safe exposure	Requires individualized adjustment	Preferred when surgeon's comfort is reduced with standard mirror setup	[1,7,11]
Mirror-image French / Lloyd-Davies technique	Surgeon between the legs; assistants laterally	Mirrored bilateral working ports with umbilical camera and epigastric/subxiphoid access	Often provides a more direct working angle for right-handed surgeons	Improved ergonomics; less instrument crossing	Requires familiarity with French positioning	Useful in technically demanding or ergonomically challenging cases	[6,11]
Three-port laparoscopic technique	Usually surgeon on patient's right	Umbilical camera, epigastric/subxiphoid working port, and left subcostal working port	Can favor selected surgeons, especially if adapted to personal dominance	Fewer incisions; simplified access	Reduced retraction and triangulation	Selected uncomplicated cases in experienced hands	[7,11]
Single-incision laparoscopic cholecystectomy (SILC/SILS)	Variable, usually central or right-sided	Single multichannel transumbilical port	Technically demanding; crowding of instruments	Single hidden incision; cosmetic benefit	Loss of triangulation; advanced skill required	Highly selected patients managed by expert minimally invasive surgeons	[17]
ICG fluorescence-assisted laparoscopic cholecystectomy	Can be combined with mirror-image American or French setups	Standard mirror-image trocar layout plus near-infrared capability	Does not change hand dominance, but improves anatomic confidence	Real-time biliary mapping; may facilitate the identification of the cystic duct and the common bile duct	Requires fluorescence technology; not universally available	Helpful when anatomy is unclear or inflammation increases dissection risk	[2]
Checkpoint-guided safe cholecystectomy strategy	Variable according to the chosen mirror setup	Uses any standard mirrored trocar pattern	Focused on anatomic orientation rather than a specific hand configuration	Emphasizes Rouvière's sulcus, hepatocystic triangle clearance, cystic plate exposure, and the critical view of safety	Not a distinct port system; it depends on the surgical discipline and experience	Recommended for all cases, especially acute inflammation or uncertain anatomy	[16,18]

In our case, a mirror-image American configuration with modified trocar placement was used to accommodate the reversed anatomy. This approach preserved adequate instrument triangulation, provided optimal exposure of the hepatocystic triangle, and allowed laparoscopic cholecystectomy to be completed without conversion to open surgery or intraoperative complications. These findings are consistent with systematic reviews emphasizing that ergonomic adaptation is more important than adherence to a specific surgical configuration and that most patients with SIT can be managed successfully using a laparoscopic approach when appropriate preoperative planning is undertaken [6,11].

Ergonomic adaptation represents one of the greatest technical challenges, particularly for right-handed surgeons, because the mirror-image anatomy alters the usual vectors of traction and dissection. Several authors have recommended modifying trocar placement and operating room positioning to facilitate dissection with the dominant hand while minimizing surgeon fatigue. In our procedure, redistribution of the trocars successfully reproduced the principles of conventional laparoscopic cholecystectomy, enabling safe dissection and optimal exposure of the biliary structures [1,16].

Regardless of trocar configuration, the primary objective remains achieving the critical view of safety before dividing any biliary or vascular structure. Systematic identification of the hepatocystic triangle, Rouvière's sulcus, and other anatomical landmarks remains the most effective strategy for preventing bile duct injury, even in the presence of mirror-image anatomy. In our patient, the critical view of safety was successfully achieved through conventional dissection, allowing clear identification of the cystic duct and cystic artery before clip application [15,16].

Indocyanine green fluorescence cholangiography is a valuable adjunct for biliary identification in patients with complex anatomy. Although not routinely required, it may be particularly useful in cases of severe inflammation or uncertain biliary anatomy. Similarly, bailout procedures, including subtotal cholecystectomy or conversion to open surgery, should be considered whenever safe anatomical identification cannot be achieved, prioritizing the prevention of bile duct injury over laparoscopic completion of the procedure [2,16].

The outcomes observed in our patient are consistent with the available literature, demonstrating that laparoscopic cholecystectomy can be performed safely in patients with SIT when supported by timely diagnosis, meticulous preoperative planning, and strict adherence to the universal principles of safe cholecystectomy. The absence of conversion to open surgery, intraoperative complications, and postoperative morbidity is consistent with the major systematic reviews, further supporting that ergonomic adaptation, rather than any specific surgical technique, is the principal determinant of successful outcomes [6,11].

Although the combination of SIT, acute biliary pancreatitis, and acute calculous cholecystitis remains uncommon, this case demonstrates that early recognition of the anatomical variation, accurate interpretation of imaging studies, and careful laparoscopic planning allow the benefits of minimally invasive surgery to be maintained without compromising patient safety [19].

## Conclusions

Acute biliary pancreatitis associated with calculous cholecystitis in patients with SIT represents an uncommon presentation of biliary disease that may delay diagnosis due to atypical pain localization and mirror-image anatomy. Early recognition through imaging studies is essential to confirm visceral inversion, define disease severity, and optimize surgical planning. SIT is not a contraindication for laparoscopic cholecystectomy; however, it requires meticulous preoperative planning, precise understanding of reversed anatomy, and adaptation of the surgical strategy, including operating room positioning, mirror-image trocar placement, and ergonomics adjusted to the surgeon's hand dominance. Regardless of the technique used, procedural success depends on careful dissection, adequate exposure of the hepatocystic triangle, and systematic achievement of the critical view of safety prior to clipping any ductal or vascular structure.

This case demonstrates that, even in the presence of rare anatomical variations and complex biliary pathology, such as concomitant acute biliary pancreatitis and calculous cholecystitis, laparoscopic cholecystectomy can be performed safely and effectively with timely recognition of the anatomical anomaly, appropriate preoperative planning, and strict adherence to safe cholecystectomy principles. The critical view of safety remains essential for preventing bile duct injury, regardless of anatomical variation.
